# Identification of HMGB2 associated with proliferation, invasion and prognosis in lung adenocarcinoma via weighted gene co-expression network analysis

**DOI:** 10.1186/s12890-022-02110-y

**Published:** 2022-08-12

**Authors:** Xie Qiu, Wei Liu, Yifan Zheng, Kai Zeng, Hao Wang, Haijun Sun, Jianhua Dai

**Affiliations:** 1 Department of Cardiothoracic Surgery, The First People’s Hospital of Lianyungang, Lianyungang, People’s Republic of China; 2 Department of Thoracic Surgery, Haian People’s Hospital Affiliated to Nantong University, Haian, People’s Republic of China; 3 Department of Cardiothoracic Surgery, The Second Affiliated Hospital of Nantong University, Nantong, People’s Republic of China; 4 Department of Thyroid Surgery, The Eighth Affiliated Hospital, Sun Yat-Sen University, Shenzhen, 518000 China; 5 Yancheng TCM Hospital, Nanjing University of Chinese Medicine, Yancheng, 224002 China

**Keywords:** HMGB2, LUAD, Biomarker, Bioinformatics, WG

## Abstract

**Background:**

High mobility group protein B2 (HMGB2) is a multifunctional protein that plays various roles in different cellular compartments. Moreover, HMGB2 serves as a potential prognostic biomarker and therapeutic target for lung adenocarcinoma (LUAD).

**Methods:**

In this study, the expression pattern, prognostic implication, and potential role of *HMGB2* in LUAD were evaluated using the integrated bioinformatics analyses based on public available mRNA expression profiles from The Cancer Genome Atlas and Gene Expression Omnibus databases, both at the single-cell level and the tissue level. Further study in the patient-derived samples was conducted to explore the correlation between HMGB2 protein expression levels with tissue specificity, (tumor size-lymph node-metastasis) TNM stage, pathological grade, Ki-67 status, and overall survival. In vitro experiments, such as CCK-8, colony-formation and Transwell assay, were performed with human LUAD cell line A549 to investigate the role of HMGB2 in LUAD progression. Furthermore, xenograft tumor model was generated with A549 in nude mice.

**Results:**

The results showed that the HMGB2 expression was higher in the LUAD samples than in the adjacent normal tissues and was correlated with high degree of malignancy in different public data in this study. Besides, over-expression of HMGB2 promoted A549 cells proliferation and migration while knocking down of HMGB2 suppressed the tumor promoting effect.

**Conclusions:**

Our study indicated that HMGB2 was remarkably highly expressed in LUAD tissues, suggesting that it is a promising diagnostic and therapeutic marker for LUAD in the future.

**Supplementary Information:**

The online version contains supplementary material available at 10.1186/s12890-022-02110-y.

## Introduction

Lung cancer is the most commonly diagnosed cancer worldwide, accounting for the greatest number of deaths among those caused by cancer [1]. The most common histological subtype of lung cancer is lung adenocarcinoma (LUAD), which accounts for more than 40% of the total lung cancer cases [2]. AlthoughLUAD can be diagnosed at an early stage using computerized tomography, many patients with non-small cell lung cancer (NSCLC) are diagnosed at an advanced stage. In recent decades, therapies, including surgery, chemotherapy, radiotherapy, molecular-target therapy, and immune therapy, have been used to improve the survival of patients with LUAD [3]. However, the prognosis of patients with advanced-stage LUAD remains grim [4, 5]. The discovery of aberrant gene expression may contribute to the early screening of patients with lung cancer and may increase the percentage of cancer diagnosis in patients at an early stage [6, 7]. Therefore, it is important to identify a novel molecular target to facilitate the early diagnosis and treatment of patients with lung cancer.

The HMGB2 protein, which belongs to the high-mobility group box (HMGB) family, which plays an essential role in transcription, chromatin remodeling, and other processes by binding to single-stranded DNA [8, 9]. *HMGB2* promotes the progression of breast cancer by targeting lactate dehydrogenase B and febrile convulsions 1 proteins [10]. In gastric cancer, *HMGB2* indicates a poor prognosis [11, 12] and is involved in tumor progression along with microRNAs, long non-coding RNAs, and proteins [13–15]. *HMGB2* is also considered as an oncogene in NSCLC and is involved in the chemotherapeutic drug resistance of NSCLC [16–18]. However, the involvement of HMGB2 in LUAD and the proliferation and invasion of LUAD cells have yet to be thoroughly investigated.

Therefore, in this study, a bioinformatics approach was employed to identify the HMGB2 as a potential diagnostic and prognostic marker for LUAD. Subsequently, the expression analysis of the HMGB2 gene in a cohort of patients with was analyzed to investigate the correlation between the *HMGB2* gene expression and the clinical characteristic of patients with LUAD.

## Methods

### Data acquisition and processing

The gene expression profiles and invasion scores of the single LUAD dataset EXP0068 were downloaded from the CancerSEA database (http://biocc.hrbmu.edu.cn/CancerSEA/home.jsp) [19, 20]. Then, the datasets GSE10072 [21], GSE21933 [22], and GSE32863 [23] were downloaded from the Gene Expression Omnibus (GEO) database (https://www.ncbi.nlm.nih.gov/geo/). The TCGA-LUAD dataset was downloaded from The Cancer Genome Atlas (TCGA) database (https://portal.gdc.cancer.gov/), and the unit of gene expression was converted from count to transcripts per kilobase million (TPM). The proteomic data of patients with LUAD (PDC000219) was downloaded from The National Cancer Institute’s Clinical Proteomic Tumor Analysis Consortium (CPTAC) (https://pdc.cancer.gov) [24]. The signature gene list for the poor survival of LUAD was downloaded from the MSigDB database [25, 26]. The signature gene list for the invasion was downloaded from the CancerSEA database. Survival data and invasion scores for each of the LUAD samples in TCGA-LUAD, GSE10072, GSE21933, and GSE32863 datasets were quantified using single-sample gene set enrichment analysis (ssGSEA) method with corresponding signature gene patterns using the R package “GSVA”.

### Weighted correlation network analysis (WGCNA)

Weighted correlation network analysis (WGCNA) was performed using the R package “WGCNA” with the protein-coding genes (PCGs) expression profiles of a single LUAD cell. In this study, the following parameters were used; soft-threshold β = 5, min module size = 30, and threshold to merge similar modules = 0.25. Pearson’s correlation analysis was performed to identify the features related modules (coefficient > 0.5, *P* < 0.05). Hub genes in the features-related module were identified according to the Pearson’s correlation between the gene expressions profiles and feature scores (coefficient > 0.3, *P* < 0.05) and module (coefficient > 0.8, *P* < 0.05).

### Protein–protein interaction (PPI) analysis

STRING database (https://www.string-db.org/) [27] and Cytoscape software were used to construct the protein–protein interaction (PPI) network according to the instructions provided on their official websites.

### Gene ontology (GO) analysis

Gene Ontology (GO) analysis was performed using “ClueGo” in Cytoscape software or Database for Annotation, Visualization, and Integrated Discovery (DAVID) v6.8 with default parameters.

## Survival analysis

Kaplan–Meier analysis of patients with LUAD based on the HMGB2 expression was performed using Gene Expression Profiling Interactive Analysis 2.0 (GEPIA 2.0) database (http://gepia2.cancer-pku.cn/#index) [28] and PrognoScan database (http://dna00.bio.kyutech.ac.jp/PrognoScan/index.html) [29].

### Functional state analysis

Patients with LUAD were divided into two groups based on the median expression of HMGB2, and GSEA analysis was performed on the data from TCGA-LUAD and GSE10072 datasets. The functional state analyses of HMGB2 in the LUAD samples at a single cell level were performed using the CancerSEA database. The TISIDB (http://cis.hku.hk/TISIDB/index.php) database [30] was used to evaluate the expression profiles of the *HMGB2* gene among the different immune subtypes of LUAD (from C1 to C6).

### Patient specimens and tissue microarray (TMA) preparation

Formalin-fixed paraffin-embedded tissue blocks, including LUAD and adjacent normal lung tissues, were collected from the The First People's Hospital of Lianyungang. The samples, collected from 2010 to 2015, were obtained from the patients with primary LUAD, who received no chemotherapy or radiotherapy. For the tissue microarray (TMA) construction, all the specimens were re-evaluated and checked by hematoxylin and eosin (HE) staining and the representative areas were selected and prepared into 1.5-mm-thick tissue cores. In this study, a total of 98 LUAD samples with adjacent normal tissues were analyzed. The clinical information of patients, including age, tumor size, Ki-67 status, lymph node status, TNM stage, pathologic grades, and follow-up information for calculating overall survival (OS) rates, were retrieved from the patients’ electronic medical records (Additional file 1: Table S1). Clinicopathological classification and staging were performed according to the 8th edition of the American Joint Committee on Cancer (AJCC) staging system. The study was reviewed and approved by the Ethics Committee of The First People's Hospital of Lianyungang. The approved number is KY-20190927005. In this study, written informed consent has been obtained from each subject and that all experiments conform to the Declaration of Helsinki.

### Immunohistochemistry (IHC)

For immunohistochemistry (IHC) analysis, 3 µm-thick TMAs slides were dewaxed in xylene and rehydrated in graded ethanol solutions. Antigens were retrieved using the high-pressure heat method with a citrate solution (pH = 6). Then, the slides were incubated with goat serum in a 3% hydrogen peroxide solution for 15 min at room temperature. The samples were then incubated with HMGB2 monoclonal antibody (1:300, Abcam, ab124670) at 4 °C overnight, which was then followed by detection with a universal SP kit (mouse/rabbit streptavidin–biotin detection system, ZSBIO, Cat # SP-9000), following the manufacturer’s instructions. The sections were then stained with 3,3-diaminobenzidine (DAB), counterstained with hematoxylin, dehydrated with a graded alcohol series, cleared in xylene, and mounted by neutral resins.

### Interpretation and evaluation of IHC results

To analyze IHC expression results, the TMA slides were scanned under an Olympus optical microscope. The HMGB2-positive stains were mainly concentrated in the nucleus; for expression analysis, both the nuclear-positive staining intensity and percentage of positive cells were graded and multiplied to obtain the overall staining score Staining intensity was scored on a scale of 0–3 as follows; 0 (negative), 1 (weak), 2 (medium), and 3 (strong). The percentage of positive tumor cells was categorized into five semi-quantitative classes: 0 (≤ 5% positive cells), 1 (6–25% positive cells), 2 (26–50% positive cells), 3 (51–75% positive cells), and 4 (> 76% positive cells). An overall staining score of > 6 was defined as the high HMGB2 expression. The expression score of HMGB2 was analyzed by two independent experienced pathologists.

### Cell culture

HMGB2-coding lentivirus vector and HMGB2 knocking-down shRNA vector combined with psPAX and Pmd2.0G were transinfected into HEK293T cells. Lentivirus was collected to infect A549 cells after 48 h. All cells were cultured in DMEM medium (Gibco) with 10% fetal bovine serum (FBS) (Gibco) and 1% penicillin–streptomycin (Gibco) at 37 °C with 5% CO_2_.

### Western blotting

Cells were lysed by pre-cooled RIPA lysis buffer (Sigma) and cell proteins were extracted.The total protein concentration was measured by bicinchoninic acid protein (BCA) assay kit (Sigma) and 10 ug total protein lysate per sample was separated via SDS-PAGE and transferred to PVDF membranes (Millipore). Membranes were blocked by skim milk for 1 h and then proteins were detected by incubating with primary antibodies HMGB2 (1:300, Abcam, ab124670) and HRP Goat Anti-Rabbit IgG (H + L) (Abcam, Cambridge, UK). Housekeep gene β-Actin (Proteintech, 20,536–1-AP, 1:5000) was used as a loading control. https://www.ncbi.nlm.nih.gov/pmc/articles/PMC7738851/-B25.

### Cell growth assay

Cells were plated into 96-well micro-plate (2000 cells/well, 3 parallel wells) at 37 °C and 5% CO_2_. Then, the cells were collected at 0 h, 24 h, 36 h, 48 h, 72 h, and cell number was analyzed by using the CCK8 reagent (MCE, HY-K0301)according to manufacturer's instruction. The optical density (450 nm) was used to indicate the number of A549 cells.

### Colony-forming assay

Dissociated cells were plated into 6-well plate (200 cells/well, 3 parallel wells). After 3 weeks, colonies were fixed with 4% paraformaldehyde for 30 min and then stained with 0.1% crystal violet solution. Each well was counted for colony-forming under a microscope.

### Transwell assay

Appropriately 5 × 10^4^cells were plated in the top chamber of Transwell (Costar, Cambridge, MA, USA) in serum-free DMEM and DMEM containing 10% FBS was added to the lower chamber. After incubation for 36 h at 37 °C, migrated cells were fixed with 4% paraformaldehyde for 30 min and then stained with 0.1% crystal violet solution.After the non-migrated cells present on the upper surface were removed, each Transwell membrane was photographed and cells were counted.

### Cell cycle assay

A549 cells were cultured in 6 wells plate for 12 h. Next, cells were digested with trypsin and suspended into single cell, and fixed with 70% ethanol at 4 °C overnight. Then cells were re-suspended and washed by PBS. 0.2 mg/mL RNase A and 100 μg/mL Propidium in PBS was applied for staining cells for 30 min. The percentages of cells of G0/G1, S, and G2/M were analyzed by counted cell numbers according to red fluorescence emission by Beckman Cytoflex.

### Animal experiment

A total of 1 × 10^6^ A549 cells infected with HMGB2-shRNA and Scramble-shRNA were injected into the subcutaneous fat tissue of nude mice aged 6 weeks purchased from Model Animal Research Center of Nanjing University. There were 5 mice in each group and all the operations in accordance with ARRIVE guidelines and animal healthcare of Nanjing Medical University discipline. Tumor volume was calculated using the formula (length × width^2^)/2. At the end of the experiment, the mice were sacrificed by Carbon dioxide gas anesthesia, and tumor masses were separated and recorded by photograph. The animal procedures were approved by the Nanjing Medical University Health Science Center Institutional Animal Care and Use Committee.

### Statistical analysis

Two-tailed t test was utilized to analyze the difference between two groups. Log-rank test was utilized for survival analysis.

## Results

### Construction of WGCNA and identification of key genes

**Fig. 1:**
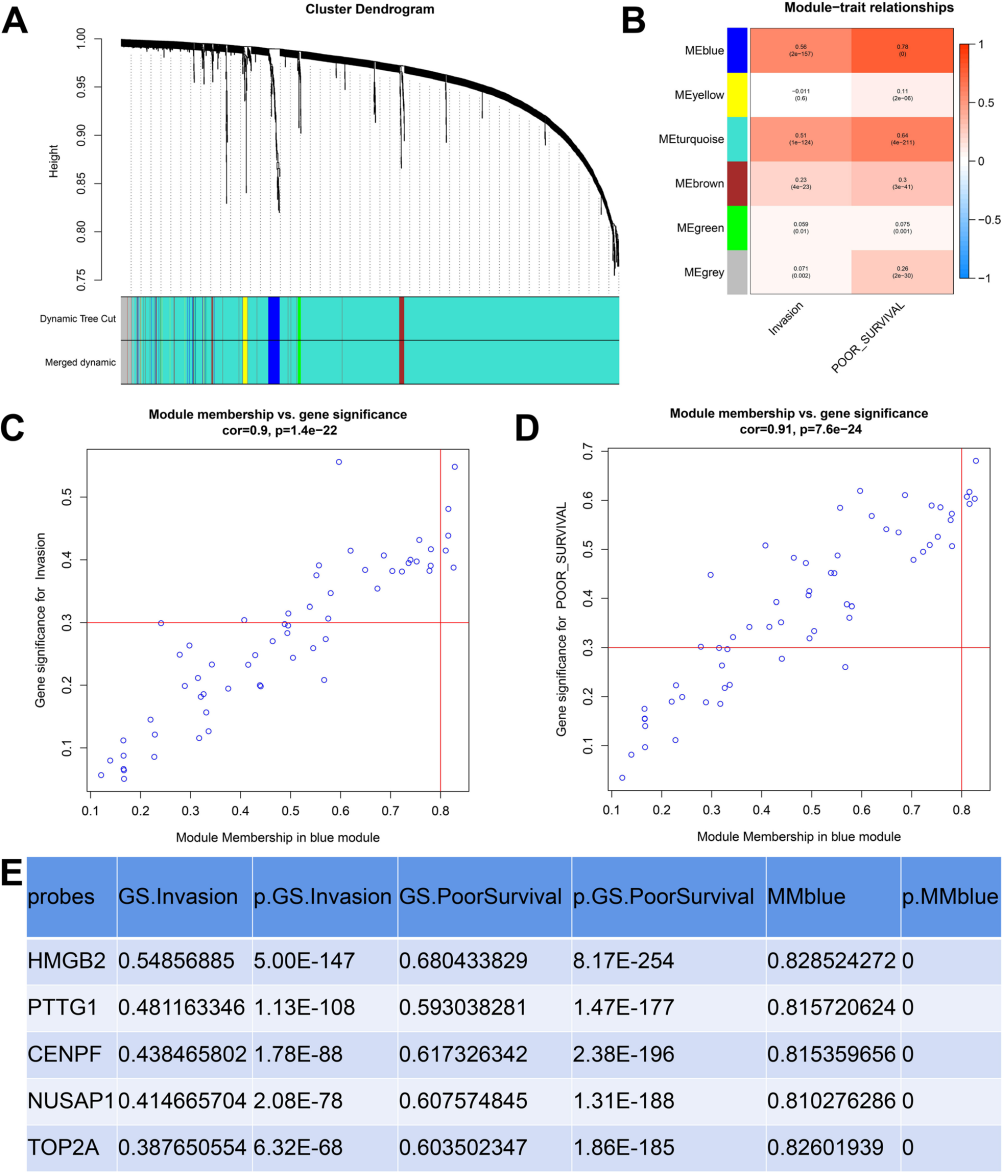
Identification of modules and genes associated with LUAD traits. **A** Dendrogram of co-expressed clusters. Each color band represents a module. **B** Heatmap of the correlation between the module and LUAD traits. **C** Screening genes with GS for invasion >  = 0.3, MM in blue module >  = 0.8. **D** Screening genes with GS for poor survival >  = 0.3, MM in blue module >  = 0.8. **E** Genes associated with LUAD traits in the blue module. GS, gene significance; MM, module membership

WGCNA was performed using the PCGs expression profile of a single LUAD dataset after quality control. A total of 6 modules were identified based on the average hierarchical clustering and dynamic tree clipping methods (Fig. 1A). The blue module was the most closely related to the invasion score and poor survival (Fig. 1B). Similarly, the genes (*HMGB2*, *PTTG1*, *CENPF*, *NUSAP1,* and *TOP2A*) in the blue module, which were closely related to the invasion and poor survival, were identified among the hub genes (Fig. 1C–E).

### PPI network and GO analysis of genes in the blue module

**Fig. 2:**
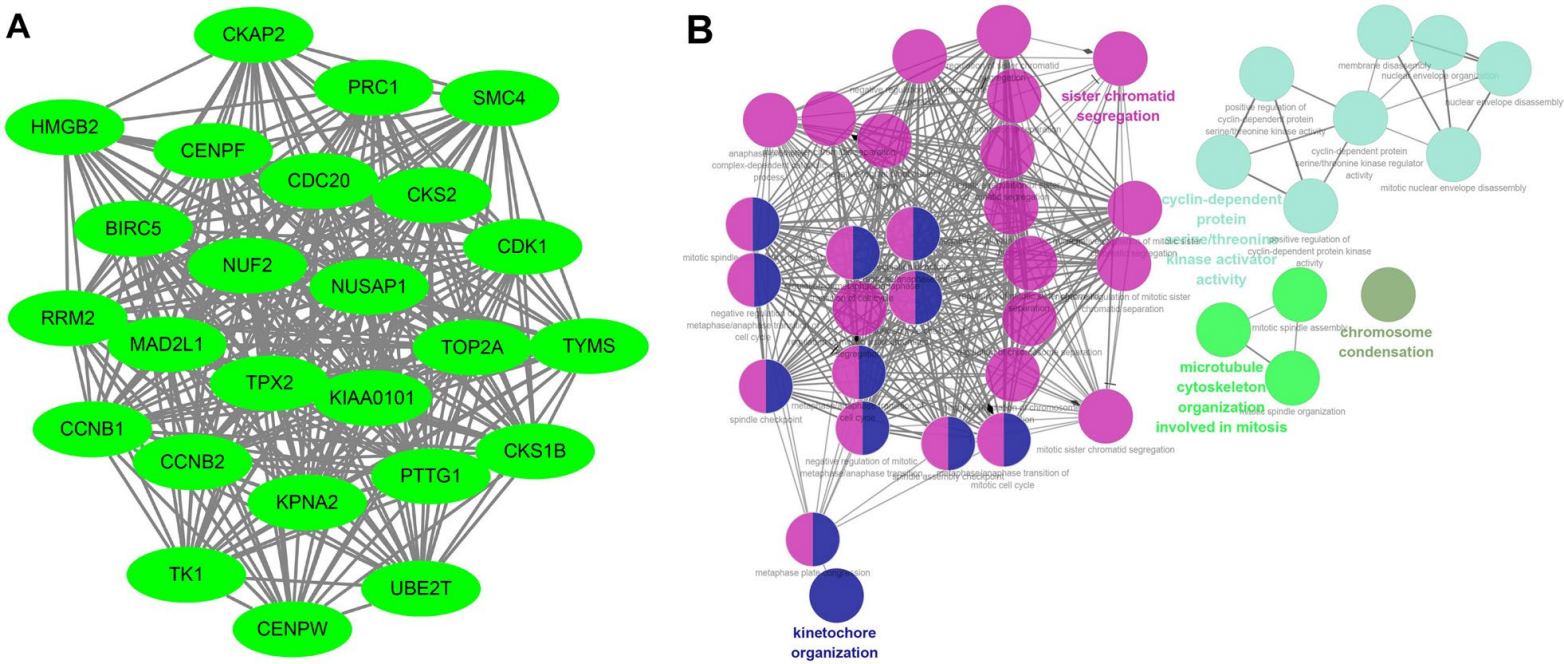
PPI network and GO annotations of the hub genes in the blue module. **A** MCODE analysis of all the 60 genes in the blue module resulted in the PPI network of 25 hub genes. **B** GO annotations of 25 hub genes in the blue module. PPI, protein–protein interaction; GO, gene ontology

The blue module consists of 60 genes, which are listed in Table 1. The PPIs among these 60 genes were identified using the STRING database. A PPI network was constructed using “MCODE” in Cytoscape software, which consisted of 25 genes (*CDC20, TOP2A, NUSAP1, PTTG1, RRM2, TK1, PRC1, CENPF, CKS2, TYMS, HMGB2, KPNA2, CKAP2, CENPW, UBE2T, SMC4, TPX2, KIAA0101, MAD2L1, CCNB2, NUF2, CCNB1, BIRC5, CKS1B,* and *CDK1*) and illustrated (Fig. 2A). The GO analysis of 25 genes using ClueGo showed that these genes might participate in the segregation of sister chromatid, cyclin-dependent protein serine/threonine kinase activator activity, chromosome condensation, organization of microtubule cytoskeleton involved in mitosis (Fig. 2B).

**Table 1:** List of genes in the blue module

Genes	GS.Invasion	p.GS.Invasion	GS.POOR_SURVIVAL	p.GS.POOR_SURVIVAL	MMblue	p.MMblue
HP1BP3	0.194489465	2.36E–17	0.342049134	2.48E–52	0.375415927	1.72E–63
HMGN2	0.248015567	1.54E–27	0.392491809	9.85E–70	0.429403445	1.51E–84
H2AZ1	0.556301554	5.23E–152	0.619374063	5.2E–198	0.596681948	2.81E–180
TXNDC12	0.087574798	0.000152652	0.155133526	1.63E–11	0.166013358	5.41E–13
HMGB2	0.54856885	5E–147	0.680433829	8.17E–254	0.828524272	0
PTTG1	0.481163346	1.13E–108	0.593038281	1.47E–177	0.815720624	0
LMNA	0.085663156	0.000212437	0.111125441	1.5E–06	0.227541962	2.5E–23
CENPF	0.438465802	1.78E–88	0.617326342	2.38E–196	0.815359656	0
MAD2L1	0.43155391	1.81E–85	0.58602481	1.99E–172	0.75751139	0
CDC20	0.416711313	3.05E–79	0.5066494	3.58E–122	0.780645386	0
CNIH4	0.115705602	5.43E–07	0.185395548	6.97E–16	0.317357887	6.58E–45
LBR	0.295566173	6.5E–39	0.414605908	2.2E–78	0.495050243	6.9E–116
RRM2	0.270310779	1.37E–32	0.482672635	1.92E–109	0.464027189	3.2E–100
NUSAP1	0.414665704	2.08E–78	0.607574845	1.31E–188	0.810276286	0
DTYMK	0.126552657	4.17E–08	0.22417539	1.13E–22	0.335943941	1.97E–50
LSM3	0.181587012	2.73E–15	0.263558096	5.23E–31	0.320540308	7.93E–46
ANP32E	0.414532352	2.36E–78	0.568420259	4.41E–160	0.61992395	1.85E–198
SMC4	0.406941975	2.6E–75	0.61080064	3.85E–191	0.686571747	3.45E–260
PRC1	0.400037124	1.3E–72	0.589546434	5.46E–175	0.739849565	5.92878775009496e–323
CCNB1	0.397426228	1.32E–71	0.525596988	5.64E–133	0.752110706	0
BIRC5	0.394914249	1.2E–70	0.508903547	2.02E–123	0.736089122	4.45089906500295e–318
UBE2T	0.391091811	3.3E–69	0.585210459	7.7E–172	0.55671854	2.79E–152
HNRNPH1	0.079826757	0.000559412	0.081351724	0.000436945	0.139160814	1.59E–09
CANX	0.050491299	0.029225736	0.139841541	1.32E–09	0.166869243	4.09E–13
TPX2	0.390565821	5.2E–69	0.572889036	3.81E–163	0.780337799	0
LSM5	0.185672298	6.3E–16	0.217785631	1.85E–21	0.326003446	1.98E–47
H2AZ2	0.232757918	2.31E–24	0.342232892	2.17E–52	0.415424258	1.02E–78
TOP2A	0.387650554	6.32E–68	0.603502347	1.86E–185	0.82601939	0
RAD21	0.199951614	2.85E–18	0.351562846	2.23E–55	0.438644081	1.49E–88
CKAP2	0.383987637	1.41E–66	0.54116512	2.23E–142	0.649091595	1.46E–223
TUBB4B	0.243836908	1.2E–26	0.333798418	8.95E–50	0.504846153	3.53E–121
CCDC34	0.211582621	2.56E–20	0.299505525	5.85E–40	0.314867874	3.38E–44
MSRB2	– 0.056602615	0.014495592	–0.034170132	0.1401855	– 0.121155745	1.54E–07
NUF2	0.382601836	4.5E–66	0.560125366	1.61E–154	0.777739445	0
HNRNPH3	0.064298362	0.005473179	0.096709601	2.87E–05	0.166721726	4.29E–13
BUB3	0.297794076	1.67E–39	0.471913779	4.72E–104	0.488204343	2.73E–112
PTMS	0.145072376	3.09E–10	0.189829616	1.37E–16	0.220034372	6.98E–22
CKS1B	0.382129621	6.69E–66	0.478582912	2.27E–107	0.703647857	8.68E–279
CCNB2	0.381374568	1.26E–65	0.495160248	6.03E–116	0.722727963	3.24E–301
MZT1	0.283437277	8.55E–36	0.406075716	5.72E–75	0.493581645	4.14E–115
CENPW	0.375372785	1.78E–63	0.487446587	6.75E–112	0.551660618	5.29E–149
CDK1	0.354106101	3.28E–56	0.534635613	2.25E–138	0.67376856	4.57E–247
HMGB3	0.346876287	7.28E–54	0.38390452	1.51E–66	0.580098229	3.46E–168
TUBA1C	0.325429384	2.93E–47	0.451808378	1.76E–94	0.53847834	1.01E–140
ARL6IP1	0.208283525	1E–19	0.260398287	2.77E–30	0.567205804	2.94E–159
UBB	0.19887289	4.34E–18	0.188569008	2.18E–16	0.288429248	4.64E–37
CALM2	0.314837801	3.45E–44	0.318958225	2.28E–45	0.495383249	4.59E–116
SKA2	0.198230093	5.58E–18	0.277240152	2.93E–34	0.440151637	3.21E–89
DCAF7	0.066230214	0.004217493	0.154166518	2.18E–11	0.166071026	5.31E–13
DDX5	0.299252295	6.83E–40	0.199262962	3.73E–18	0.241041764	4.63E–26
KPNA2	0.273618228	2.22E–33	0.388151454	4.12E–68	0.570266281	2.42E–161
TK1	0.156623239	1.04E–11	0.296986593	2.74E–39	0.331512416	4.43E–49
CKS2	0.306621658	6.84E–42	0.360689519	2.11E–58	0.57519621	9.58E–165
TYMS	0.263405175	5.67E–31	0.447817381	1.17E–92	0.29786502	1.6E–39
SNX5	0.121354136	1.47E–07	0.223181068	1.76E–22	0.228520426	1.61E–23
PCLAF	0.304101046	3.36E–41	0.507961995	6.72E–123	0.407520658	1.54E–75
EIF2S2	0.111905656	1.27E–06	0.175157876	2.57E–14	0.165517307	6.35E–13
DDX39A	0.233122056	1.95E–24	0.321454453	4.3E–46	0.342708414	1.54E–52
LSM4	0.248708321	1.09E–27	0.301903889	1.32E–40	0.278432804	1.49E–34
UBE2S	0.259184683	5.23E–30	0.451218342	3.28E–94	0.545264365	6.14E–145

*GS* gene significance, *MM* module membership

### mRNA expression of HMGB2 was higher in LUAD tissues than in the normal lung tissues

**Fig. 3:**
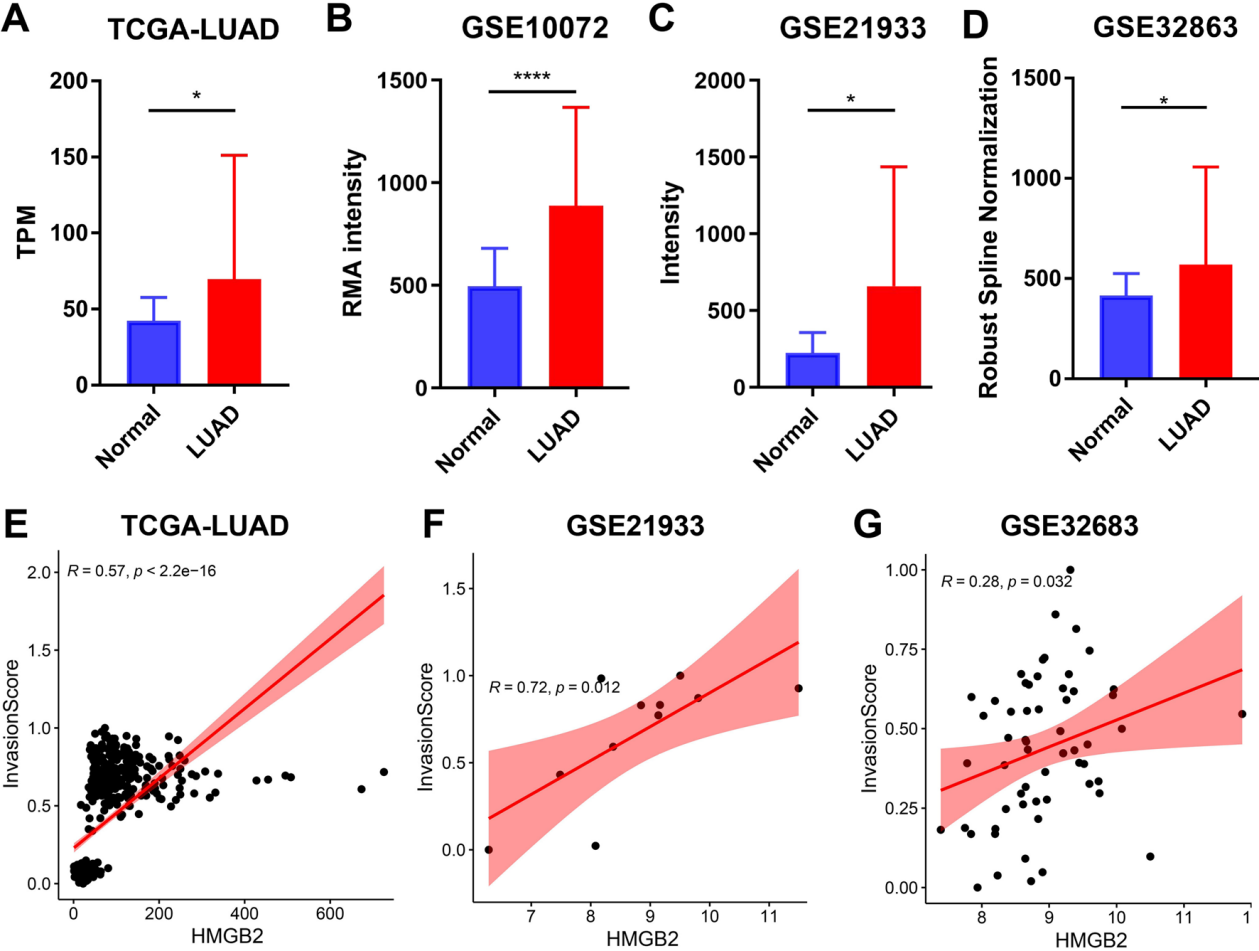
High expression of *HMGB2* was correlated with invasion in LUAD. **A**–**D** *HMGB2* mRNA expression was higher in the LUAD than in normal lung samples in **A** TCGA-LUAD, **B** GSE10072, **C** GSE21933, **D** GSE32863 datasets. **E**–**G** *HMGB2* expression was positively correlated with the invasion score of LUAD samples in **E** TCGA-LUAD, **F** GSE21933, and **G** GSE32863 datasets. P* < 0.05, P** < 0.01, P**** < 0.0001

The mRNA expression of the *HMGB2* gene was compared between the LUAD and normal lung tissues using four public datasets. Both the TCGA-LUAD dataset (Fig. 3A) and three GEO datasets (GSE10072, GSE21933, and GSE32863) (Fig. 3B–D) showed that the mRNA expression of the *HMGB2* gene was higher in the LUAD tissues than in that in the normal lung tissues. Besides, the mRNA expression of the *HMGB2* gene was positively correlated with the invasion score of LUAD samples in the TCGA-LUAD, GSE21933, and GSE32863 datasets (Fig. 3E–G).

### High mRNA expression of HMGB2 predicted poor prognosis of patients with LUAD

**Fig. 4:**
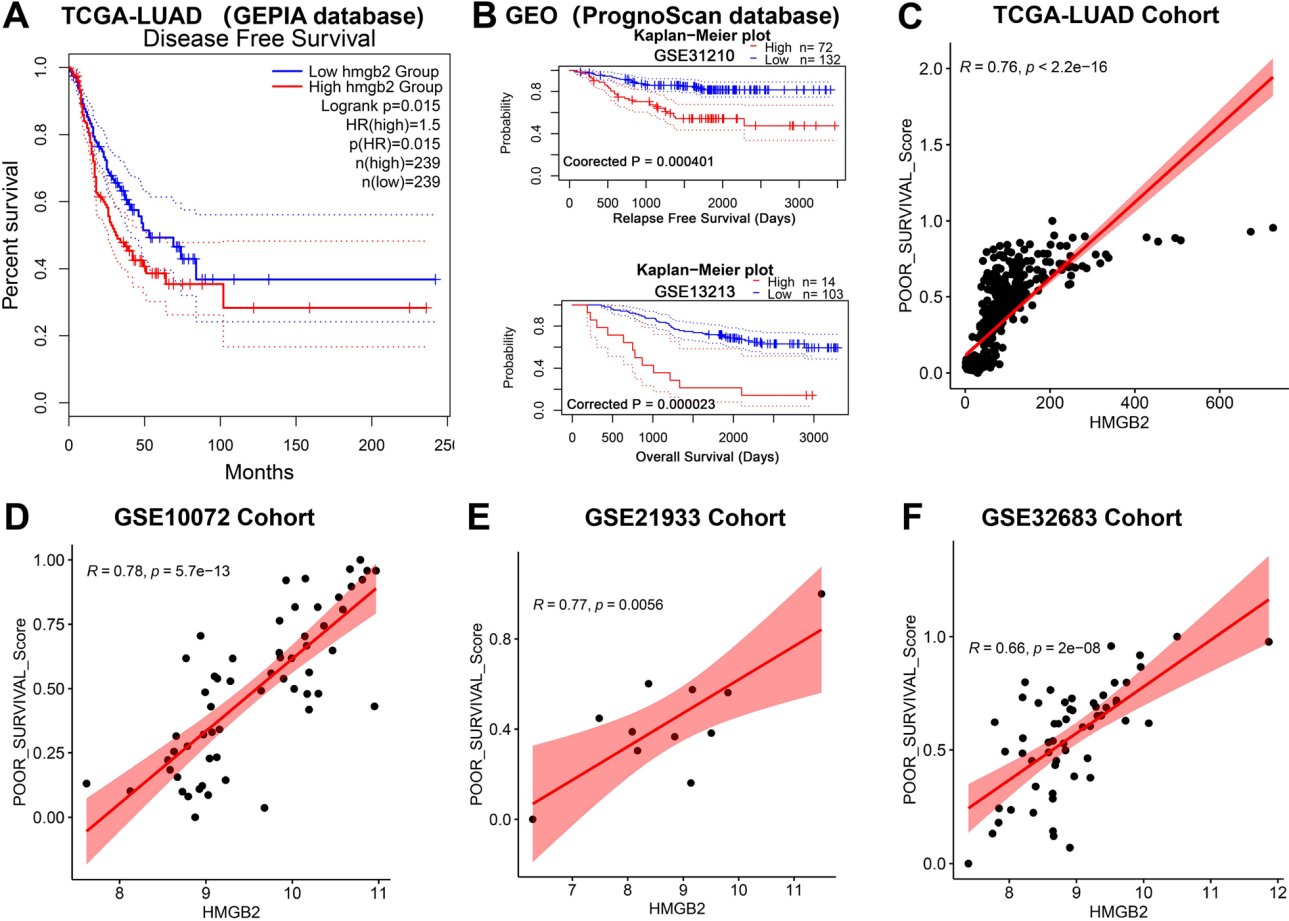
High *HMGB2* expression predicted poor prognosis of the patients with LUAD. **A** Patients with the high *HMGB2* expression had shorter DFS than those with the low *HMGB2* expression. **B** Patients with the high *HMGB2* expression had shorter RFS and OS than those with the low *HMGB2* expression. **C**–**F** *HMGB2* expression was positively correlated with the poor survival of LUAD samples in **C** TCGA-LUAD, **D** GSE10072, **E** GSE21933, and **F** GSE32863 datasets. DFS, disease-free survival; RFS, relapse-free survival; OS, overall survival

First, using the GEPIA online tool, the patients with LUAD with high *HMGB2* expression were found to exhibit shorter disease-free survival (DFS) than that those with low *HMGB2* expression (Fig. 4A). Next, the PrognoScan database demonstrated that the patients with LUAD with high *HMGB2* expression had shorter relapse-free survival (RFS) and OS than that those with low *HMGB2* expression (Fig. 4B). Additionally, it was also found that the mRNA expression of the *HMGB2* gene was positively correlated with the poor survival of LUAD samples in the TCGA-LUAD, GSE10072, GSE21933, and GSE32863 datasets (Fig. 4C–F).

### Elevated HMGB2 levels were correlated with the poor prognosis of LUAD

**Fig. 5:**
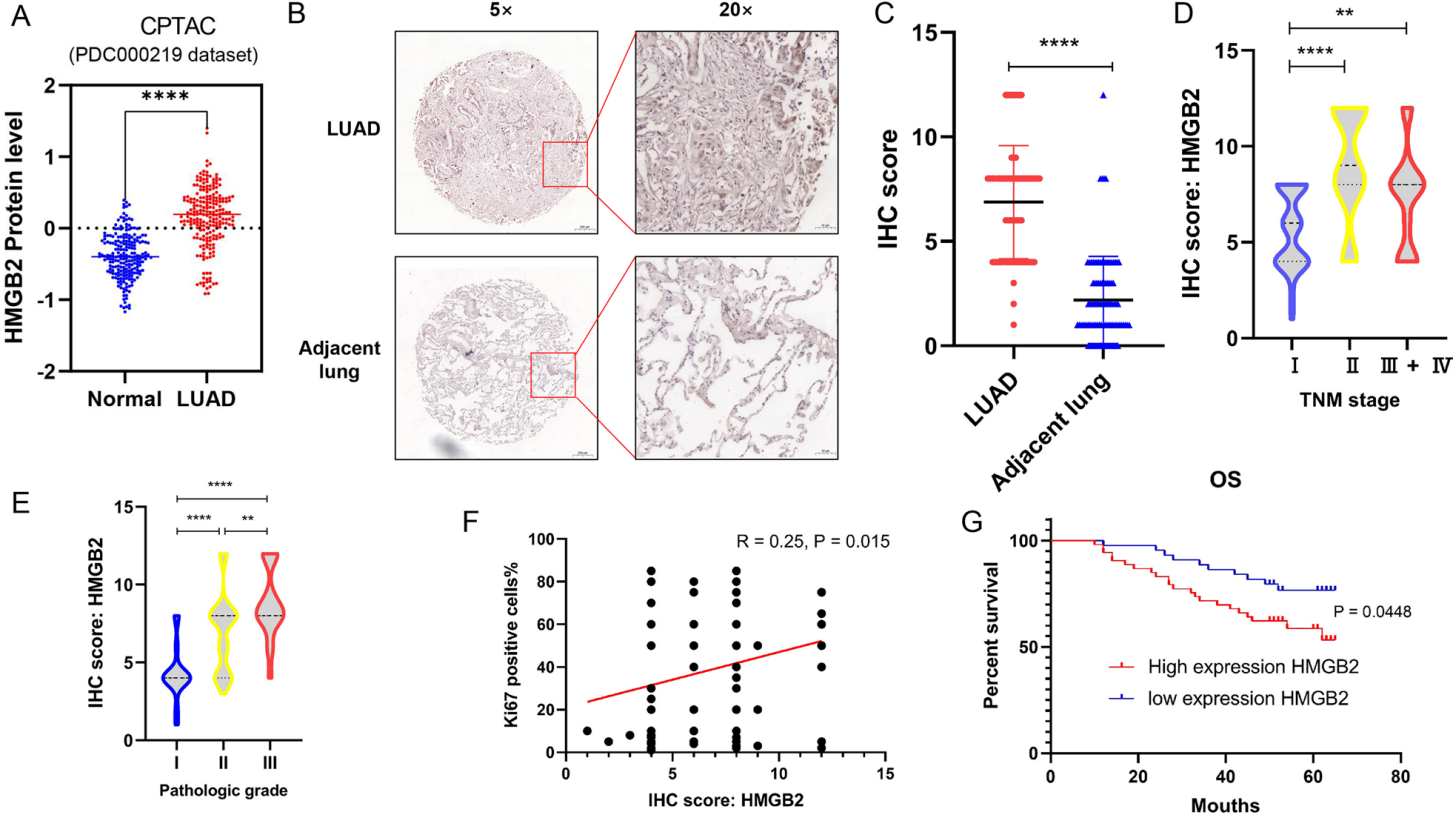
Elevated HMGB2 levels were correlated with the poor prognosis of LUAD. **A** Elevated protein expression of HMGB2 was observed in LUAD using public data from PDC000219. **B** A representative case, showing the elevated expression of HMGB2 in LUAD as compared to that in the adjacent alveolus tissue; **C** Quantitative analyses of the IHC staining of HMGB2 in the tumor sample and paired adjacent tissue of 98 patients with LUAD; **D** Quantitative analyses of the IHC staining of HMGB2 in 98 LUAD patients TMAs with different TNM-stages; **E** Quantitative analyses of the IHC staining of HMGB2 in 98 LUAD patients TMAs with different pathologic grades **F** Correlation of the IHC score of HMGB2 and percentage of Ki67 in 98 LUAD patients. **G** Kaplan–Meier analysis of the overall survival of 98 patients with HMGB2 (two groups stratified by HMGB2 expression level. Differences between the groups were shown using a log-rank test.)

Higher protein expression of HMGB2 was observed in LUAD than that in normal lung tissues using a public data from CPTAC (Fig. 5A). To identify the protein expression level of HMGB2 in LUAD tissues, an IHC assay was performed using a TMA panel, which contained the tissue samples of 98 patients with LUAD. The results comfirmed a significantly higher level of HMGB2 expression in the tumor tissues than that in the adjacent alveolar tissues (Fig. 5B and C). Moreover, the protein level of HMGB2 in the patients with advanced LUAD was remarkably higher than that in the early-stage patients (Fig. 5D). Subsequently, the upregulation of the HMGB2 gene was positively correlated with poor differentiation grade (Fig. 5E). Furthermore, the expression level of the *HMGB2* gene was positively correlated with the percentage of Ki-67-positive cells (Fig. 5F). Moreover, patients with LUAD with a high level of HMGB2 (quantitative IHC score > 6) had a shorter survival time than those with low levels of HMGB2 (Fig. 5G). In brief, these results suggested that the HMGB2 gene could be regarded as an independent prognostic marker for patients with LUAD.

### The expression level of HMGB2 was positively correlated with cell cycle and proliferation in the LUAD tissues

**Fig. 6:**
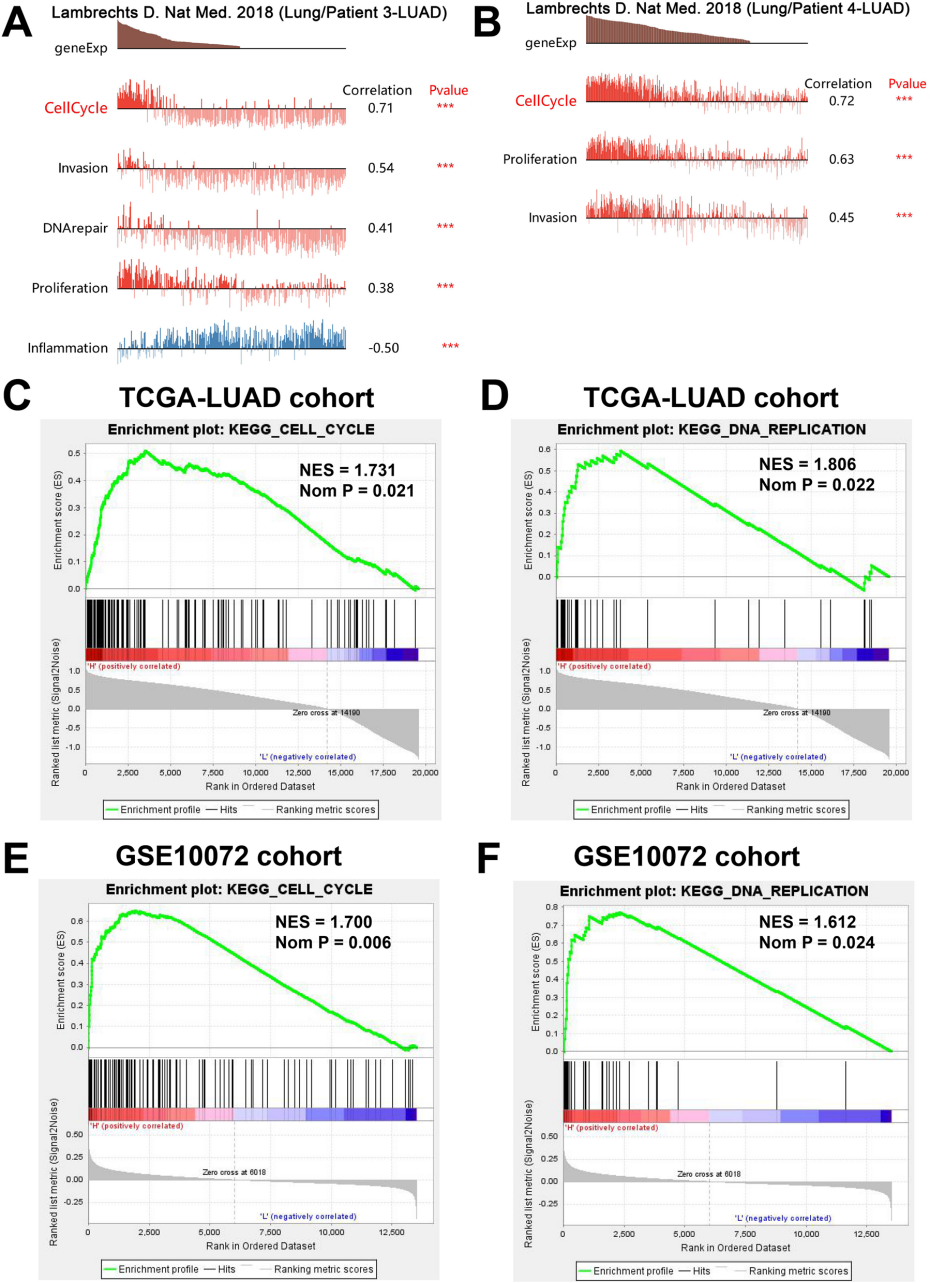
HMGB2 was correlated with cell cycle and proliferation in LUAD samples. **A** Correlations between the functional states and HMGB2 expression in patient 3 (LUAD) at the single-cell level. **B** Correlations between the functional states and HMGB2 expression in patient 4 (LUAD) at the single-cell level. **C**, **D** GSEA for HMGB2 using the data from the TCGA-LUAD dataset. **C** Cell cycle and **D** DNA replication pathways were enriched in the high expression group. **E** and **F** GSEA for HMGB2 using the data from the GSE10072 dataset. **E** Cell cycle and **F** DNA replication pathways were enriched in the high expression group

Pearson's correlation analysis employing the expression patterns of single LUAD cells from two patients with LUAD patients (Pearson's coefficient > 0.3, *P* < 0.05) revealed that HMGB2 expression was positively correlated with cell cycle, proliferation, and invasion, as evidenced by the CancerSEA database (Fig. 6A and B). Futhermore, the GSEA analysis, based on the data from TCGA-LUAD and GSE10072 datasets, showed that both the cell cycle and DNA replication pathways were significantly enriched in the LUAD samples with high *HMGB2* expression (Fig. 6C–F).

### PPI network and GO analysis of HMGB2 and co-expressed genes

**Fig. 7:**
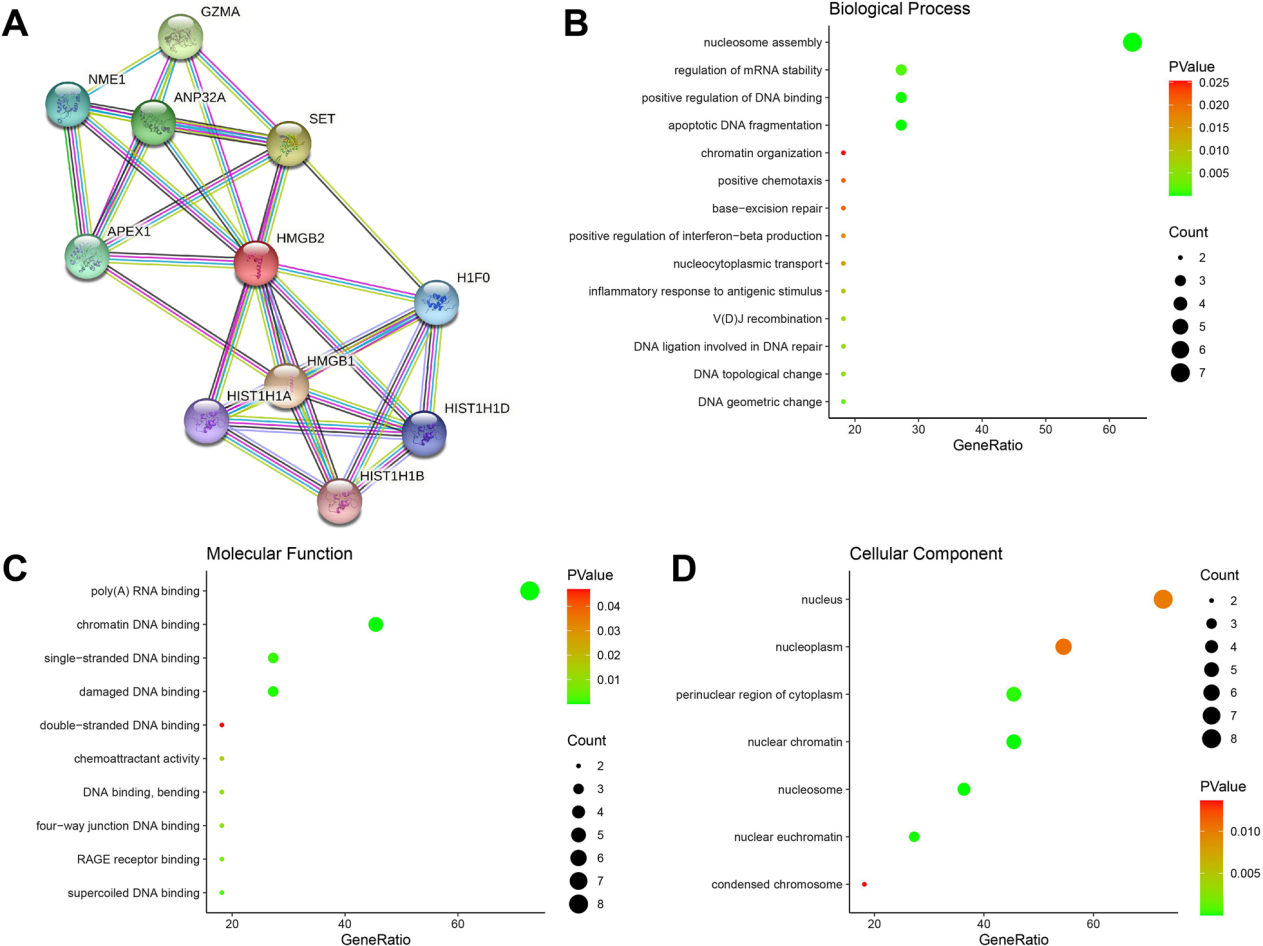
PPI network and GO annotations of HMGB2 and its co-expressed genes **A** PPI network of HMGB2 and its co-expressed genes. **B**–**D** Significant **B** biological process, **C** molecular function, and **D** cellular component from the GO annotations of HMGB2 and its co-expressed genes (*P* < 0.05). PPI, protein–protein interaction; GO, gene ontology

HMGB2 was used as the input as a single protein in the STRING database. The PPI network of HMGB2 and its co-expressed genes consisted of 11 genes (*HMGB2, HMGB1, HIST1H1A, HIST1H1B, HIST1H1D, H1F0, SET, APEX1, ANP32A, NME1,* and *GZMA*), which are shown in (Fig. 7A). The GO analysis of 11 genes using DAVID showed that these genes might participate in biological processes, such as nucleosome assembly, regulation of mRNA stability, positive regulation of DNA binding, and apoptotic DNA fragment (Fig. 7B); molecular function, such as like poly (A) RNA binding and chromatin DNA binding (Fig. 7C); and cellular component, such as nucleus and nucleoplasm (Fig. 7D).

### HMGB2 was negatively related to inflammation in the LUAD tissues

**Fig. 8:**
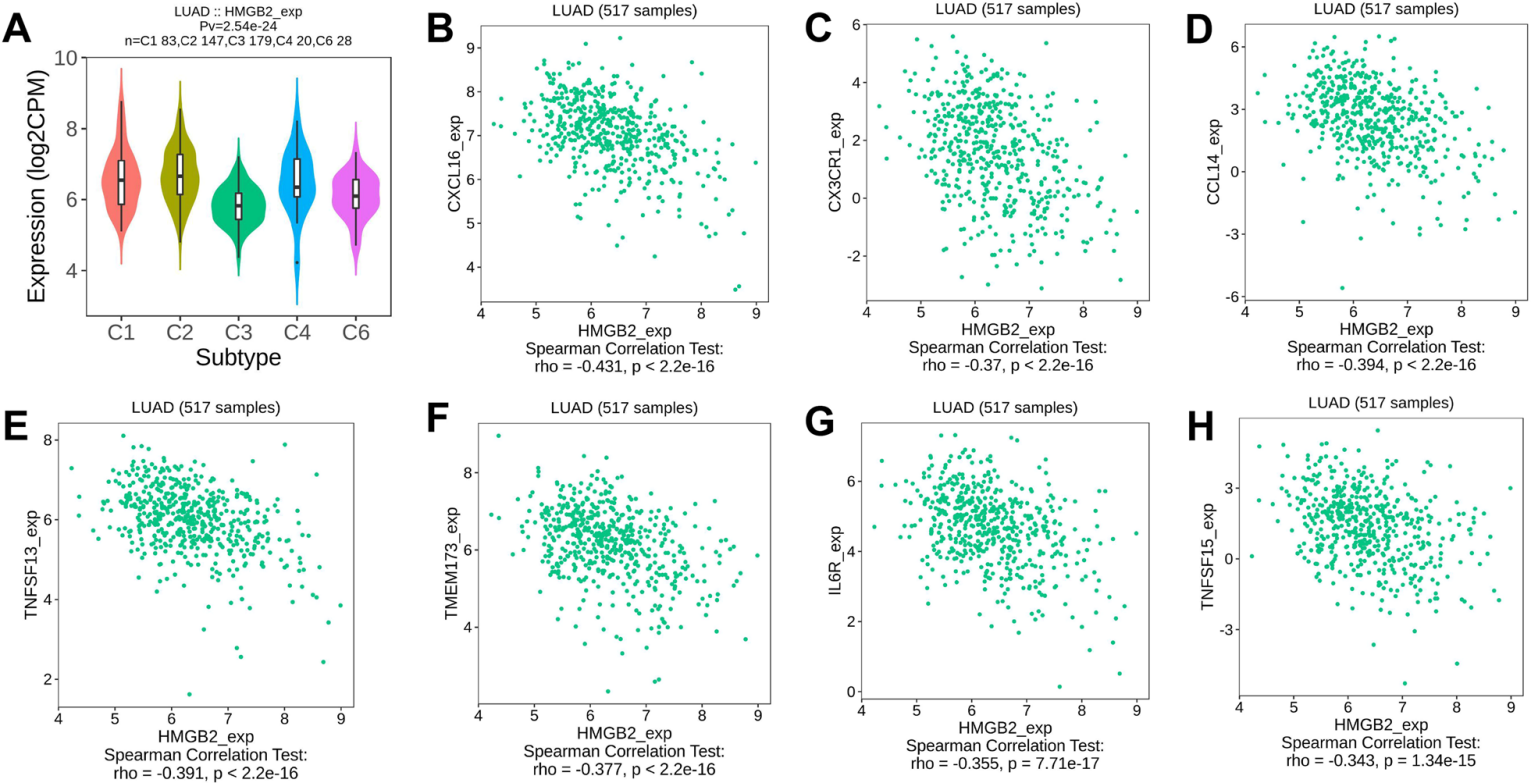
*HMGB2* expression was negatively correlated with inflammation in LUAD. **A** *HMGB2* expression among the different immune subtypes of LUAD samples. **B**–**D** *HMGB2* expression was negatively correlated with the expression of chemokines **B** *CXCL16*, **C** *CX3CR1*, and **D** *CCL14* in LUAD. **E**–**H** *HMGB2* expression was negatively correlated with the expression of immuno-stimulators **E** *TNFSF13*, **F** *TMEM173*, **G** *IL6R*, and **H** *TNFSF15* in LUAD

As shown in Fig. 6A, the *HMGB2* expression was negatively correlated with the inflammation score in LUAD patients 3 in EXP0068 cohort at the single-cell level (coefficient = -0.50 and *P* < 0.001). Furthermore, it was found that the C3 immune type (inflammatory) of patients with LUAD had the lowest expression of *HMGB2* as compared to the other immune types (Fig. 8A). Results of Pearson’ correlation analysis showed that the expression of *HMGB2* was negatively correlated with that of chemokines (*CXCL16*, *CX3CR1*, and *CCL14*) (Fig. 8B–D) and immunostimulatory proteins (*TNFSF13*, *TMEM173*, *IL6R*, and *TNFSF15*) (Fig. 8E–H).

### Overexpression of HMGB2 promoted A549 cells proliferation and migration

**Fig. 9:**
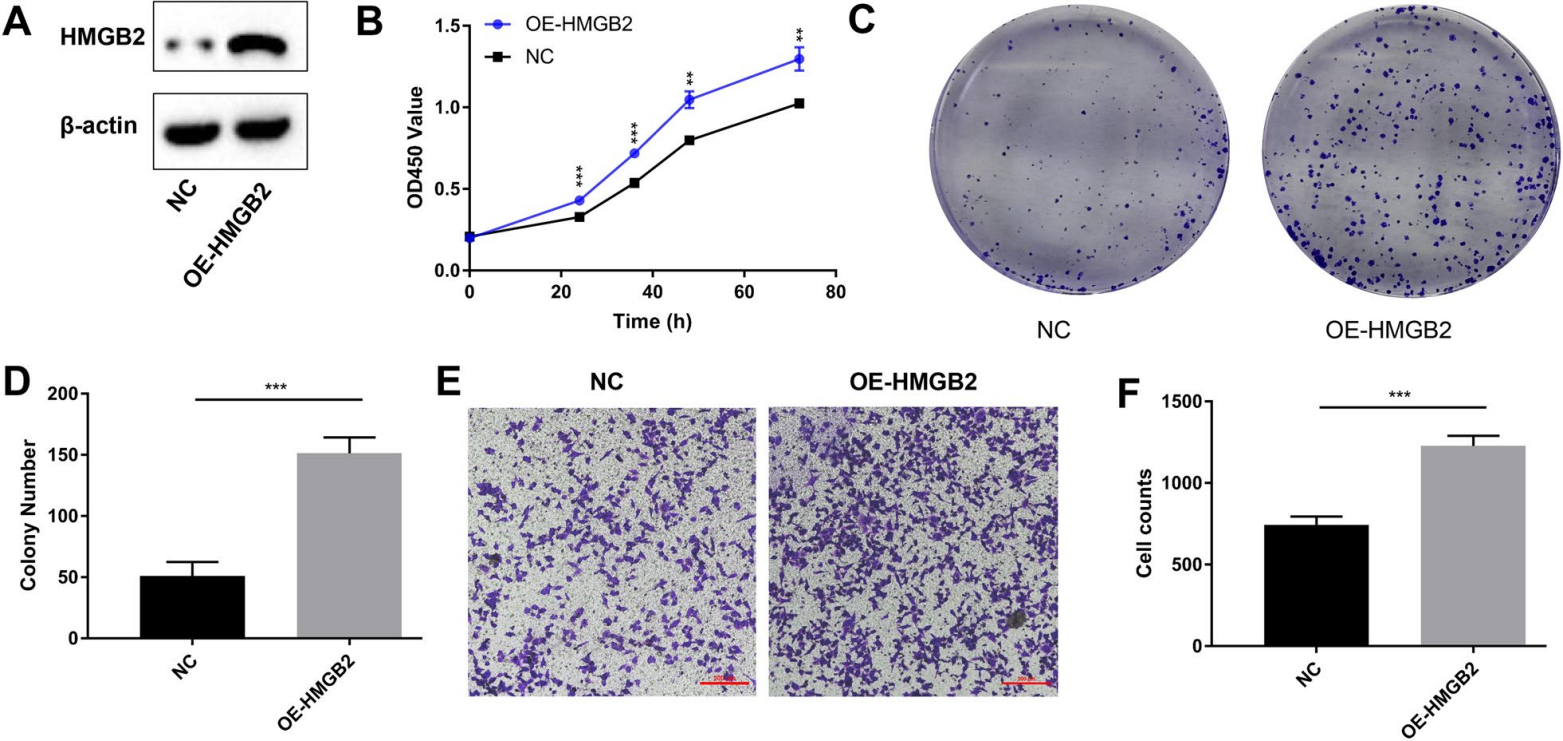
HMGB2 promoted A549 cells colony-formation and migration. **A** The protein level of HMGB2 and β-actin in A549 cells between HMGB2 over-expression group (OE-HMGB2) and negative control group (NC) were determined by western blot assay. **B** HMGB2 over-expression promoted A549 cells growth. **C** HMGB2 over-expression promoted A549 cells colony-formation. **D** Histogram of colony numbers between HMGB2 over-expression group and control group. **E** HMGB2 over-expression promoted A549 cells migration. **F** Histogram of cell counts between HMGB2 over-expression group and control group. ***P < 0.001

HMGB2 stable expression A549 cell line was constructed and validated as shown in Fig. 9A and Additional file 2; Figure S1. The in vitro experiments’ results showed that over-exprssion of HMGB2 promoted A549 cells proliferation (Fig. 9B) and colony-formation (Fig. 9C and D). Besides, Transwell assay’ result showed that HMGB2 may promote the ability of A549 cells to migrate (Fig. 9E and F).

### Knockdown of HMGB2 inhibited A549 cells proliferation, tumorigenicity and migration

**Fig. 10:**
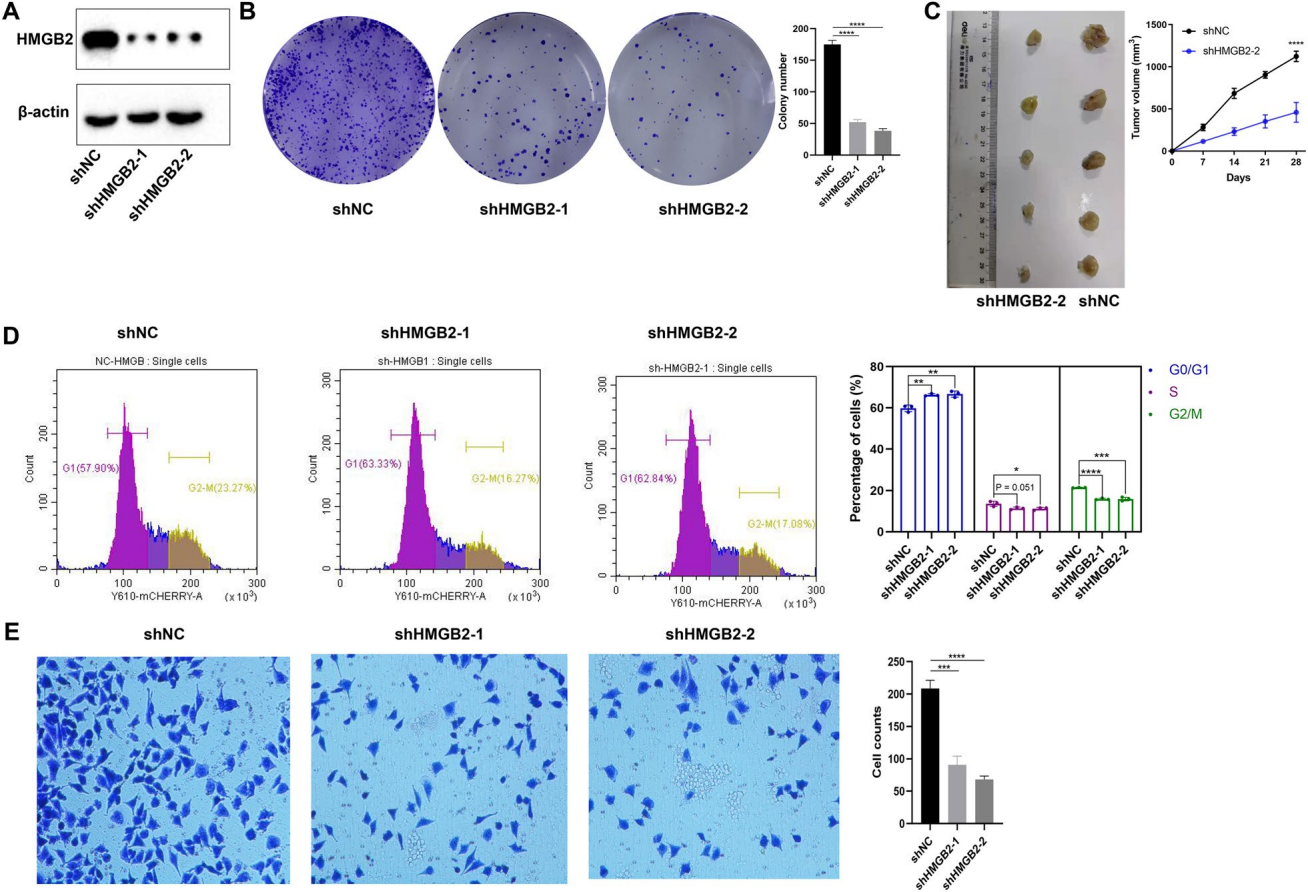
Knockdown of HMGB2 inhibited A549 cells colony-formation, tumorigrnicity and migration. **A** The protein level of HMGB2 and β-actin in A549 cells between HMGB2 knockdown group (shHMGB2-1 and shHMGB2-2) and shRNA negative control group (shNC) were determined by western blot assay. **B** Knockdown of HMGB2 inhibited A549 cells colony-formation. **C** Knockdown of HMGB2 inhibited A549 cells tumor volume in nude mouse model. **D** Knockdown of HMGB2 increased the number of A549 cells in the G0/G1 phase and decreased the number of cells in the G2/M phase. **E** Knockdown of HMGB2 inhibited A549 cells migration. *P < 0.05; **P < 0.01; ***P < 0.001; ****P < 0.0001

HMGB2 knockdown A549 cell line was constructed and validated as shown in Fig. 10A. The colony assays showed that knockdown of HMGB2 inhibited the colony-formation of A549 cells (Fig. 10B). The in vivo experiments showed that knockdown of HMGB2 inhibited the volumes of the tumors (Fig. 10C). Cell cycle assay showed that the knockdown of HMGB2 increased the number of cells in the G0/G1 phase and decreased the number of cells in the G2/M phase (Fig. 10D). Besides, we observed that knockdown of HMGB2 could inhibited the migration of A549 cells (Fig. 10E).

## Discussion

Extensive efforts have been devoted to exploring the promising diagnostic and therapeutic target for improving the prognosis of lung cancer. In this study, we performed WGCNA and ssGSEA analyses with the expression profiles of patients with LUAD at a single-cell level and identified the *HMGB2* gene was identified as a promising diagnostic and prognostic biomarker for the LUAD (Additional file 2; Figure S2). Furthermore, using an integrated bioinformatics analysis on multiple expression profiles from TCGA and GEO datasets, we found that the HMGB2 might affect the prognosis of patients with LUAD patients by regulating the proliferation and invasion of LUAD cells.

HMGB2, a member of the family of high mobility group nonhistone chromatin proteins, regulates the processes of transcription, replication, recombination, and DNA repair [31]. HMGB2 is highly expressed during embryogenesis, however, its expression is limited in the adult organs and is mainly detected in lymphoid organs and testes. However, previous studies have elaborated on the elevated expression of HMGB2 in several types of tumor tissues and reported it as an oncogene. In breast cancer, the HMGB2 is regulated with ER, LDHB, and FBP1 to promote the endocrine therapy resistance and tumorigenesis of tumor cells [10, 32, 33]. In gastric cancer, the high expression of HMGB2 predicts a poor prognosis [11, 34] and its expression is regulated by non-coding RNA, miRNA-23b-3p, miRNA-1297, MALAT1, and miRNA-873 to promote the proliferation, migration, and invasion of cells [13–15]. In prostate cancer, the early detection of HMGB2 in prostate tissues using IHC contributed to the early-stage diagnosis of prostate cancer [35]. In cervical cancer, pancreatic cancer, glioma, and ovarian cancer, the HMGB2 was reported as a reliable prognosis predictor. In NSCLC, the high expression of HMGB2 in cancer was associated with the chemotherapy response and poor prognosis [36, 37]. Together with PDIA3, p21, and LINC00184, the HMGB2 can regulate the chemotherapy- and radiotherapy-induced DNA damage to enhance drug resistance [17, 38, 39]. All these results indicated that the HMGB2 might play an important role in the development of tumors, including lung cancer. However,no evidence has demonstratedits effect on tumor growth in NSCLC, especially in LUAD.

In this study, HMGB2 was screened as a promising biomarker for LUAD. Furthermore, the integrated bioinformatics analysis, using the data from public platforms both at tissue and single-cell levels indicated that the HMGB2 might affect the cell cycle, proliferation, and expression of inflammatory factors in LUAD. For further validation, the correlation of HMGB2 with clinical characteristics was analyzed using TMA staining for HMGB2 on 98 LUAD specimens. The results showed that the expression of HMGB2 was dramatically elevated in tumor cells as compared to that in the normal alveolar cells. Moreover, the HMGB2 expression level was identified to be highly correlated with a poor TNM stage, pathologic grade, and prognosis. Noteworthily, the expression of HMGB2 was positively correlated with that of Ki67 in LUAD. In conclusion, this study demonstrated that HMGB2 is a potential diagnostic and therapeutic indicator for LUAD, suggesting that HMGB2 might be a potential therapeutic target in LUAD.

## Conclusions

We propose that HMGB2 may be correlated with proliferation of LUAD cells and it is a promising diagnositic and therapeutic marker for LUAD.

## Supplementary Information

**Additional file 1: Table S1.** Clinical informations and IHC score of patients with LUAD.

**Additional file 2: Figure S1.** Original blot and images. **Figure S2.** HMGB2 expression is not correlated with survival of patients with LUSC.

## Data Availability

The main results of this study are available from public databases, such as CancerSEA database (http://biocc.hrbmu.edu.cn/CancerSEA/home.jsp), GEO database (https://www.ncbi.nlm.nih.gov/geo/), TCGA database (https://portal.gdc.cancer.gov/), MSigDB database (http://www.gsea-msigdb.org/gsea/index.jsp), STRING database (https://www.string-db.org/), GEPIA2.0 database (http://gepia2.cancer-pku.cn/#index), PrognoScan database (http://dna00.bio.kyutech.ac.jp/PrognoScan/index.html), TISIDB database (http://cis.hku.hk/TISIDB/), and CPTAC database (https://pdc.cancer.gov). The clinical information and IHC data for human specimens were shown in Additional file 1: Table S1.

## Abbreviations

LUAD: Lung adenocarcinoma
NSCLC: Non-small cell lung cancer
HMGB2: High-mobility group box 2
TCGA: The cancer genome atlas
GEO: Gene expression omnibus
ssGSEA: Single-sample gene set enrichment analysis
WGCNA: Weighted correlation network analysis
PPI: Protein–protein interaction
TMA: Tissue microarray
OS: Overall survival
IHC: Immunohistochemistry
