# Correction: Identification of HMGB2 associated with proliferation, invasion and prognosis in lung adenocarcinoma via weighted gene co-expression network analysis

**DOI:** 10.1186/s12890-022-02170-0

**Published:** 2022-10-13

**Authors:** Xie Qiu, Wei Liu, Yifan Zheng, Kai Zeng, Hao Wang, Haijun Sun, Jianhua Dai

**Affiliations:** 1 Department of Cardiothoracic Surgery, The First People’s Hospital of Lianyungang, Lianyungang, People’s Republic of China; 2 Department of Thoracic Surgery, Haian People’s Hospital Affiliated to Nantong University, Haian, People’s Republic of China; 3 Department of Cardiothoracic Surgery, The Second Affiliated Hospital of Nantong University, Nantong, People’s Republic of China; 4 Department of Thyroid Surgery, The Eighth Affiliated Hospital, Sun Yat-Sen University, 518000 Shenzhen, China; 5 Yancheng TCM Hospital, Haijun Sun, Nanjing University of Chinese Medicine, 224002 Yancheng, China

BMC Pulmonary Medicine (2022) 22:310

10.1186/s12890-022-02110-y

Following publication of the original article [1], the authors provided the following corrections:

1) In the Keyword section, “WG” should be “WGCNA.” 

2) In the Authors’ contributions section, “YZ: performed the experiments and collected the breast cancer samples” should be “YZ: performed the experiments and collected the LUAD cancer samples” (Page 14, Right column, line 8). 

3) In the Funding section, “This research was supported by Youth Elite Project of The First People’s Hospital of Lianyungang [QN1903 ]” should be “This research was supported by Youth Elite Project of The First People’s Hospital of Lianyungang [QN1903] and Scientific Research Project of Nantong Municipal Health Committee to Yifan Zheng (MA2020002).”

The original article has been updated accordingly. The authors apologize for any inconvenience caused and thank you for reading this correction.

