# Supplementary material for: Identification of HMGB2 associated with proliferation, invasion and prognosis in lung adenocarcinoma via weighted gene co-expression network analysis
Source: BMC Pulm Med. 2022 Aug 12;22:310. doi: 10.1186/s12890-022-02110-y (PMC9373369; doi:10.1186/s12890-022-02110-y)

Identification of HMGB2 Associated with Proliferation, Invasion and Prognosis in Lung Adenocarcinoma via Weighted Gene Co-Expression Network Analysis

Xie Qiu[1]*, Wei Liu[2]*, Yifan Zheng[3]*, Kai Zeng[4], Hao Wang[5], Haijun Sun[1#], Jianhua Dai[1#]

Supplementary Table Legend

Table S1. Clinical informations and IHC score of patients with LUAD

Figure S1. Original blot and images.

Figure 9A Original blot and images.

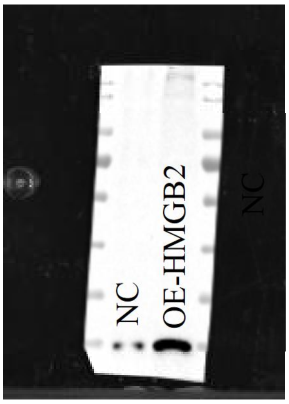

HMGB2

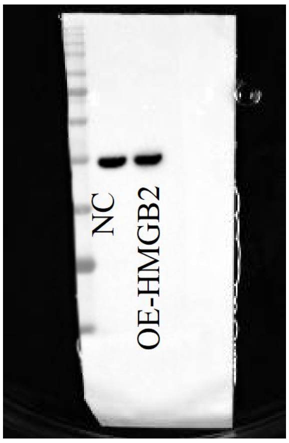

B -actin

Figure 10A Original blot and images.

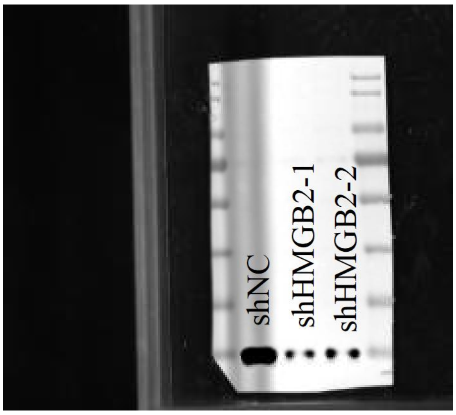

HMGB2

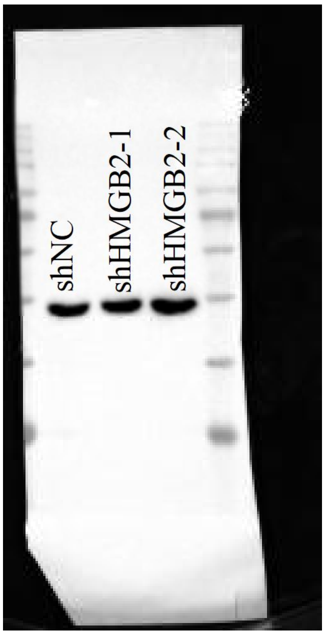

Β -actin

Figure S2. HMGB2 expression is not correlated with survival of patients with LUAD.

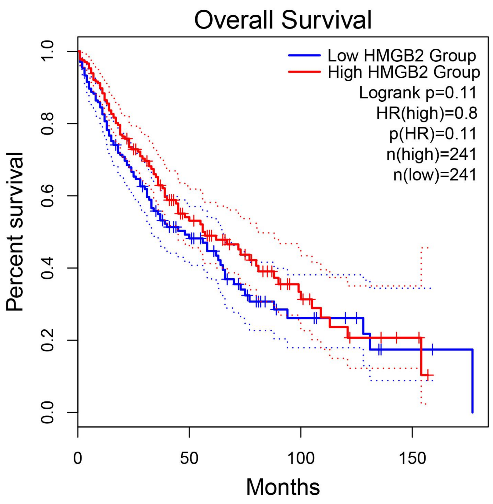

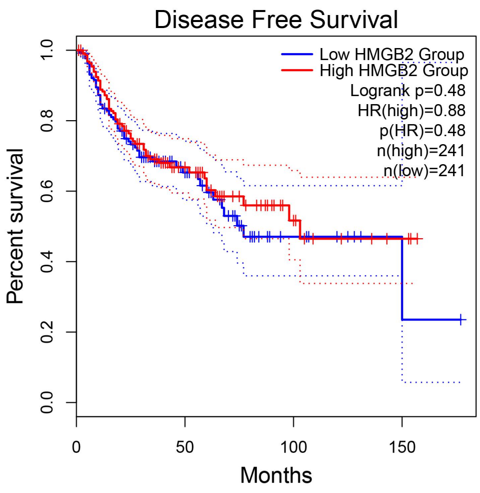

Supplement: Supplementary file 2 — Additional file 2: Figure S1. Original blot and images. Figure S2. HMGB2 expression is not correlated with survival of patients with LUSC. [file 12890_2022_2110_MOESM2_ESM.pdf]
